# The Effects of Continuous vs. Intermittent Caloric Restriction on Fat Loss: A Randomized Controlled Trial

**DOI:** 10.3390/nu18111823

**Published:** 2026-06-05

**Authors:** Kelly E. Johnson, Briana Curran, Sydney Roberson, Haley Corso, Emily Hoelscher, Bill I. Campbell, Kamryn Rabon, Amelia Lovering, Madison Albert

**Affiliations:** 1 Department of Kinesiology, Coastal Carolina University, Conway, SC 29528, USA; blcurran@coastal.edu (B.C.); saroberso@coastal.edu (S.R.); hmcorso@coastal.edu (H.C.); erhoelsch@coastal.edu (E.H.); kbrabon@coastal.edu (K.R.); amloverin@coastal.edu (A.L.); mmalbert@coastal.edu (M.A.); 2 Exercise Science & Kinesiology Program, Performance and Physique Enhancement Laboratory, University of South Florida, Tampa, FL 33620, USA; bcampbell@usf.edu

**Keywords:** obesity, body composition, weight loss, fat mass, fat-free mass

## Abstract

**Background/Objectives**: Obesity remains difficult to treat effectively, not because weight loss cannot be achieved, but because it is difficult to sustain in the face of physiological adaptations to energy restriction, including reductions in resting metabolic rate and loss of fat-free mass. Dietary strategies that preserve favorable body composition while supporting long-term adherence are therefore needed. The purpose of this study was to compare continuous caloric restriction (CCR) with an intermittent approach incorporating structured diet refeeds and planned diet breaks (DRF) on body composition outcomes in adult women with obesity. **Methods**: Thirty adult females (18–65 years; BMI 30–45 kg·m^−2^) were randomized to 12 weeks of CCR or DRF following a two-week maintenance phase used to determine individualized caloric needs. Both groups were prescribed a 25% caloric deficit and protein intake of 1.2 g·kg^−1^·day^−1^. Body composition, including body fat percentage, fat mass, and fat-free mass, was assessed using air-displacement plethysmography at baseline and post-intervention. **Results**: Repeated-measures ANOVA revealed a significant main effect of time for body fat percentage (*p* < 0.001), which decreased by 6.7 ± 2.1% in the CCR group and 6.0 ± 1.9% in the DRF group, with no significant group × time interaction (*p* > 0.05). Fat mass significantly declined in both groups (*p* < 0.001), with reductions of 9.30 ± 2.77 kg (CCR) and 9.21 ± 2.63 kg (DRF); between-group differences were negligible (*p* > 0.05; Cohen’s d = 0.03). Fat-free mass increased over time (*p* < 0.05); although the interaction was not significant (*p* = 0.08), the DRF group demonstrated a moderate effect size advantage. Despite similar changes in body composition, analysis of energy balance revealed a significantly greater daily energy deficit in the CCR group compared with DRF (−1005 ± 515 vs. −690 ± 120 kcal·day^−1^, *p* = 0.041), indicating a higher achieved level of caloric restriction in CCR. **Conclusions**: Both dietary strategies effectively reduced fat mass in females with obesity; however, incorporating diet breaks was associated with a nonsignificant trend toward greater preservation or accrual of fat-free mass without compromising fat loss. Future studies should investigate this potential association in larger, adequately powered trials before any conclusions regarding metabolic adaptation or practical advantage can be drawn.

## 1. Introduction

Obesity is characterized by an elevated risk of cardiometabolic disease, including type 2 diabetes, cardiovascular disease, hypertension, and broader metabolic dysfunction [1,2]. Clinically, relatively modest reductions in body mass (≈5–10%) are sufficient to produce meaningful improvements in cardiometabolic markers and reduce disease burden [3,4]. However, the primary limitation in obesity management is not initial weight loss, but the difficulty of sustaining it, as physiological and behavioral adaptations to caloric restriction promote weight regain over time [5,6].

Current clinical guidelines primarily advocate continuous caloric restriction (CCR), which involves a sustained daily reduction in energy intake below maintenance requirements [1,2]. Although CCR is effective for short-term weight loss, prolonged energy restriction often leads to compensatory metabolic adaptations, including reductions in resting metabolic rate, increased appetite, and unfavorable hormonal responses, which can undermine long-term success [5,7]. These challenges have driven interest in alternative dietary strategies that may improve adherence while attenuating metabolic adaptation.

Intermittent caloric restriction (ICR) encompasses dietary approaches that alternate periods of caloric restriction with periods of energy balance or increased intake. Common implementations include alternate-day fasting, the 5:2 diet, and structured diet refeeds or diet breaks. Experimental and animal models suggest that intermittent restriction may reduce fat mass and improve metabolic flexibility while limiting adaptive thermogenesis [8].

In human studies, however, outcomes comparing ICR and CCR are mixed. Short-term investigations generally report similar reductions in body weight and fat mass between approaches [9,10,11]. Some trials in overweight and obese women have demonstrated greater reductions in body fat or improvements in insulin sensitivity with intermittent restriction despite comparable weight loss [11,12]. The MATADOR study further demonstrated that intermittent energy restriction incorporating extended diet breaks resulted in greater fat loss efficiency compared with continuous restriction in obese men [13]. Large randomized trials lasting up to one year have shown comparable weight loss and cardiometabolic outcomes between ICR and CCR, suggesting that overall energy deficit may be the primary determinant of weight loss [14,15,16,17].

While weight loss magnitude may be similar, preservation of fat-free mass is a critical consideration, particularly in women with obesity. Loss of lean mass during dieting can negatively impact metabolic health, physical function, and long-term weight-maintenance capacity. Physiological adaptations to sustained caloric restriction, including persistent reductions in energy expenditure, may disproportionately affect women and increase susceptibility to weight regain [7,18].

Importantly, evidence from resistance-trained populations suggests that intermittent caloric strategies may offer unique benefits for lean mass preservation. Campbell and colleagues demonstrated that resistance-trained individuals following intermittent energy restriction with structured carbohydrate refeeds better preserved fat-free mass, dry fat-free mass, and resting metabolic rate compared with continuous caloric restriction, despite similar reductions in fat mass [18]. These findings support the hypothesis that periodic increases to maintenance energy intake may attenuate metabolic adaptation and preserve lean tissue during weight loss.

Despite growing interest in diet refeeds and diet breaks, most randomized controlled trials examining intermittent versus continuous restriction have included mixed-sex samples or male-dominant cohorts. To our knowledge no study has directly examined the effects of a structured intermittent caloric restriction strategy incorporating both diet refeeds and prolonged diet breaks exclusively in females with obesity. Therefore, the purpose of this randomized controlled trial was to compare the effects of continuous caloric restriction versus intermittent caloric restriction incorporating structured diet refeeds and diet breaks on body weight, fat mass, fat-free mass, and body fat percentage in adult females with obesity.

## 2. Methods

### 2.1. Participants

Thirty adult females were recruited from the Horry County, South Carolina area via email announcements and community outreach. Eligible participants were between 18 and 65 years of age, had a body mass index (BMI) between 30 and 45 kg·m^−2^, reported body weight stability (±4.5 kg) over the prior three months, engaged in less than 150 min of structured physical activity per week, and had access to an Apple^®^ iPhone or Android^®^ smartphone compatible with dietary and body weight tracking applications.

Exclusion criteria included engagement in >150 min of weekly physical activity; planned travel during the study; weight loss >4.5 kg within the previous three months; pregnancy or suspected pregnancy; history of bariatric surgery within the previous 12 months; major surgery within four weeks of enrollment; diagnosis of type 1 diabetes; immunodeficiency disorders; kidney disease; recent myocardial infarction (<3 months); neurological or psychiatric disorders impacting decision making; current eating disorders; use of dietary supplements known to significantly alter metabolism or body weight; or inability to attend laboratory visits between 06:00 and 12:00. Participants with known metabolic, cardiovascular, or musculoskeletal conditions contraindicating dietary intervention were also excluded.

All participants provided written informed consent prior to participation. Ethical approval was obtained from the Institutional Review Board of Coastal Carolina University [IRB #2025.23], and all procedures were conducted in accordance with the Declaration of Helsinki.

### 2.2. Study Design

**Figure 1:**
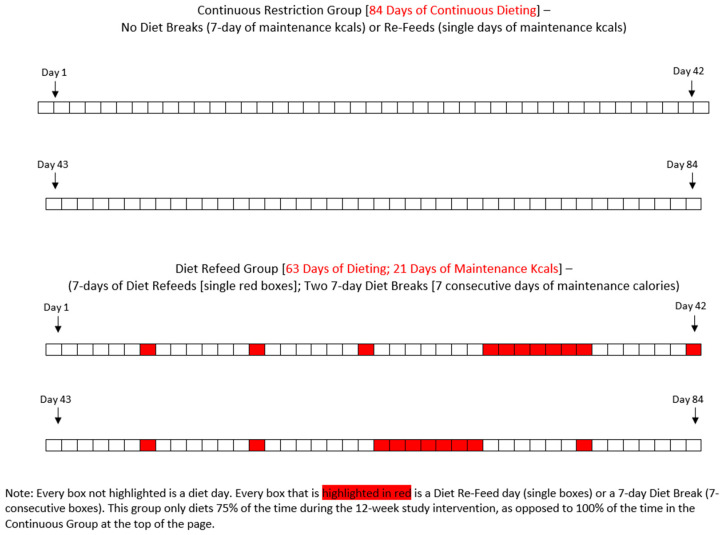
Continued calorie restriction and diet refeed protocol. Every box not highlighted is a diet day. Every box that is highlighted in red is a Diet Re-Feed day (single boxes) or a 7-day Diet Break (7-consecutive boxes). This group only diets 75% of the time during the 12-week study intervention, as opposed to 100% of the time in the Continuous Group at the top of the page.

This investigation employed a randomized, parallel-group design consisting of two phases: (1) a 2-week pre-diet maintenance phase and (2) a 12-week dietary intervention. Participants were randomly assigned, using a computer-generated randomization sequence, to one of two dietary strategies: (1) continuous caloric restriction (CCR) or (2) intermittent caloric restriction incorporating structured diet refeeds and diet breaks (DRF). Randomization was implemented to minimize allocation bias and ensure comparable baseline characteristics between groups. Participants completed laboratory visits at the Body Composition Laboratory at Coastal Carolina University, including pre-diet counseling, baseline assessment, and post-intervention testing. A schematic overview of the dietary interventions is provided in Figure 1.

The two intervention groups differed in how caloric restriction was implemented. Participants in the CCR group followed a continuous energy restriction, maintaining an approximate 25% daily caloric deficit throughout the entire 12-week intervention without interruption. In contrast, participants in the DRF group followed the same 25% daily caloric deficit but incorporated periodic increases in energy intake. Specifically, every seventh day, participants consumed calories at estimated maintenance levels (“refeed days”), and during weeks 5 and 10, they completed structured 7-day “diet breaks” during which they consumed maintenance-level calories. This design allowed for direct comparison between a traditional continuous dieting approach and an intermittent strategy that alternates periods of caloric restriction with planned periods of energy balance.

### 2.3. Pre-Diet Maintenance Phase

During the pre-diet phase (study visit #1), participants were instructed to maintain their habitual dietary intake while achieving body weight stability over a two-week period. Weight stability was defined as a body weight fluctuation of no more than ±1–2% of baseline body weight. During this time, participants attended an in-person visit for body weight assessment and individualized nutritional counseling, which included standardized instruction on portion estimation, food label interpretation, and dietary logging.

Participants were required to weigh themselves daily using a Coach Care^®^ Smart Body Analyzer scale (New York, NY, USA) synced with the CoachCare^®^ mobile application (Version 2.61.410) and to record all food and beverage intake throughout the two-week period using the CoachCare^®^ app. Daily body weight data were reviewed by the research team and used, along with dietary records, to determine individual maintenance caloric intake, defined as the habitual caloric intake at which body mass neither increases nor decreases.

If participants demonstrated consistent weight gain or loss over at least three consecutive days, they were provided with individualized instructions to adjust energy intake from the provided diet to re-establish weight stability.

All food and beverage intake was recorded daily throughout the study, including detailed documentation of food type, portion size, preparation method, and timing of intake. Nutrient composition was verified using an integrated nutrition database. Research staff reviewed dietary logs on a weekly basis to ensure accuracy, completeness, and compliance with the prescribed dietary protocol.

Maintenance caloric intake was calculated as the average daily caloric intake during the pre-diet phase in which body weight remained stable, and this value was used to guide dietary prescriptions during diet break weeks. Prior research reports, in semicontrolled free-living settings, prior research has reported that app-based tracking methods have demonstrated acceptable validity compared with weighed food intake assessments [19].

### 2.4. Dietary Intervention

Following completion of the maintenance phase, participants began a 12-week dietary intervention. Both groups were prescribed a protein intake of 1.2 g·kg^−1^·day^−1^ to support the preservation of fat-free mass during caloric restriction.

During diet break weeks, dietary intake continued to be closely monitored, and participants received ongoing feedback based on both dietary records and body weight trends. If consistent weight gain or loss was observed over a minimum of three consecutive days, research staff provided targeted dietary adjustments to maintain intended caloric targets and stabilize body weight during the diet break period.

### 2.5. Continuous Caloric Restriction Group (CCR)

Participants assigned to the CCR group consumed a daily caloric intake corresponding to a 25% reduction from their individualized maintenance calories for the entire 84-day intervention period. No planned refeeds or diet breaks were incorporated.

### 2.6. Intermittent Caloric Restriction Group (DRF)

Participants assigned to the dietary restriction with refeeds (DRF) group followed an intermittent caloric restriction protocol designed to achieve an overall caloric deficit equivalent to the continuous restriction group while incorporating structured periods of energy balance. Individual maintenance energy requirements were determined during the pre-diet phase.

During caloric restriction phases, participants consumed a daily energy intake corresponding to a 25% reduction from maintenance calories. Protein intake was prescribed at 1.2 g·kg^−1^·day^−1^ across all phases of the intervention. During restriction days, remaining energy intake was distributed to provide approximately 45–50% of total energy from carbohydrates and 25–30% from fat.

The weekly structure consisted of six consecutive days of a 25% caloric deficit followed by one day at maintenance caloric intake, referred to as a diet refeed. On refeed days, total caloric intake was increased to maintenance levels while protein intake remained constant at 1.2 g·kg^−1^·day^−1^. The additional calories relative to restriction days were primarily allocated to carbohydrates, resulting in an approximate macronutrient distribution of 55–60% carbohydrate, 20–25% fat, and protein held constant.

In addition to weekly refeeds, participants completed two planned seven-day diet breaks, implemented every third week of the intervention. During diet breaks, participants consumed maintenance calories each day while maintaining the same protein prescription. Carbohydrate intake was increased relative to restriction phases to account for the higher energy intake, while fat intake remained proportionally consistent. Following the first diet break, the cycle of caloric restriction and weekly refeeds was resumed and repeated a second time.

Across the intervention, participants in the DRF group completed 63 days of caloric restriction (71.5%) and 21 days at maintenance caloric intake (28.5%).

Participants were instructed to weigh themselves once per week using a Coach Care^®^ Smart Body Analyzer scale (New York, NY, USA) and to log all food intake throughout the intervention using the CoachCare^®^ mobile application. Dietary adherence and compliance were supported through scheduled video conference check-ins with members of the research team conducted via the CoachCare^®^ app, during which body weight trends and dietary records were reviewed.

### 2.7. Dietary Adherence and Monitoring

Dietary intake throughout the intervention was self-reported using the CoachCare^®^ mobile application (Coach Care^®^, New York, NY, USA), through which participants logged all food and beverage consumption on a daily basis. Participants were provided standardized training on dietary logging, portion estimation, and food label interpretation prior to the intervention, and adherence expectations were reinforced throughout the study.

Energy intake targets and macronutrient prescriptions were individualized based on maintenance caloric needs determined during the pre-diet phase. Protein intake targets were prescribed and reviewed during counseling sessions; however, biochemical verification of dietary intake (e.g., nitrogen balance, doubly labeled water, respiratory quotient) was not performed.

Dietary adherence was supported through scheduled weekly check-ins with members of the research team conducted via video conference using the CoachCare^®^ application. During these meetings, logged dietary intake and body weight trends were reviewed to provide feedback, clarify logging discrepancies, and reinforce compliance with prescribed energy and macronutrient targets. Formal investigator-led dietary audits and predefined quantitative compliance thresholds (e.g., ≥80% of days meeting caloric targets) were not prospectively established.

### 2.8. Body Composition Assessment

Body composition was assessed at baseline (week 0) and post-intervention (week 12), and at the three-week follow-up using air displacement plethysmography (BODPOD^®^, COSMED, Concord, CA, USA). For all assessments, participants arrived in a fasted state, wore minimal tight-fitting clothing, and a standardized nylon swim cap to reduce measurement error. All assessments were administered by the same trained technician.

Body composition outcomes included body mass (BM), body fat percentage (BF%), fat mass (FM), and fat-free mass (FFM). Two consecutive measurements were obtained during each testing session and averaged for analysis.

### 2.9. Fat-Free Mass Index Calculation

Fat Mass Index (FMI), which is a ratio expressing the amount of fat mass relative to an individual’s height squared (kg·m^−2^); the Fat-Free Mass Index (FFMI), which measures the proportion of lean body mass (muscles, bones, organs, etc.) relative to height squared; and the Fat Mass to Fat-Free Mass Ratio (FM/FFM), which compares the amount of fat mass to fat-free mass in the body.

### 2.10. Energy Balance and Estimation of Caloric Restriction Calculation

To address potential bias in self-reported dietary intake, energy balance and adherence to caloric restriction were estimated using changes in body composition, following the approach described in the CALERIE study [20].

Changes in body energy stores (ΔES) were calculated from measured changes in fat mass (FM) and fat-free mass (FFM) over the 12-week intervention. Energy equivalents of 9.3 kcal·g^−1^ for FM and 1.1 kcal·g^−1^ for FFM were applied:

ΔES = (ΔFM × 9.3) + (ΔFFM × 1.1)ΔES = (ΔFM × 9.3) + (Δ FFM × 1.1)ΔES = (ΔFM × 9.3) + (ΔFFM × 1.1) where FM and FFM were expressed in grams. Total ΔES was divided by 84 days to obtain average daily energy imbalance (kcal·day^−1^) [20].

### 2.11. Statistical Analysis

Weekly caloric intake was averaged for each participant, and mean daily energy intake across the 12-week intervention was compared between groups using independent-samples *t*-tests. Patterns of energy intake over time were analyzed using repeated-measures ANOVA with group (continuous caloric restriction [CCR] vs. diet refeed [DRF]) as the between-subjects factor and week (1–12) as the within-subjects factor, allowing evaluation of main effects of group and time, as well as group × time interactions. Macronutrient intake (protein, carbohydrate, and fat; expressed as g·day^−1^) and total fat mass loss were also compared between groups using independent-samples *t*-tests. When significant main effects or interactions were identified, post hoc comparisons were performed using Bonferroni-adjusted pairwise tests.

Changes in body weight, body fat percentage, fat mass, and fat-free mass were analyzed using repeated-measures ANOVA with time (baseline vs. post-intervention) as the within-subjects factor and group (CCR vs. DRF) as the between-subjects factor.

To provide an objective estimate of adherence to caloric restriction, changes in body energy stores (ΔES) were calculated from changes in fat mass and fat-free mass using established energy coefficients (9.3 kcal·g^−1^ for fat mass and 1.1 kcal·g^−1^ for fat-free mass). Mean daily ΔES (kcal·day^−1^) over the 12-week intervention was computed and compared between groups using independent-samples *t*-tests. This body composition–based approach provides an estimate of energy balance independent of self-reported dietary intake.

Baseline characteristics were assessed using independent-samples *t*-tests to confirm group equivalency prior to the intervention. Effect sizes for ANOVA outcomes were calculated using partial eta squared (η^2^_p_) and interpreted as small (0.01), moderate (0.06), or large (0.14), while effect sizes for between-group comparisons were quantified using Cohen’s d (0.2 = small, 0.5 = moderate, 0.8 = large).

All data are presented as mean ± standard deviation, and statistical significance was set a priori at *p* < 0.05. Statistical analyses were performed using SPSS Version 31.0 (IBM Corp., Armonk, NY, USA).

## 3. Results

Thirty participants completed the 12-week dietary intervention, with no adverse events reported. Baseline characteristics were similar between groups and are presented in Table 1. All outcome findings are presented in Table 2. Compliance with dietary intake and body-weight monitoring was high across groups, as verified through weekly dietary log reviews.

### 3.1. Body Weight and Body Fat Percentage

Participants in the continuous caloric restriction (CCR) group lost an average of 7.53 ± 1.91 kg body weight, while those in the diet refeed (DRF) group lost 6.80 ± 1.77 kg of body weight. Body weight significantly decreased over time in both groups (main effect of time, *p* < 0.001). The between-group difference in weight loss was not statistically significant (*p* > 0.05) and corresponded to a small effect size (Cohen’s d = 0.39), indicating comparable reductions in body mass between dietary strategies. A significant main effect of time was also observed for body fat percentage (*p* < 0.001). Body fat percentage decreased by 6.7 ± 2.1% in the CCR group and 6.0 ± 1.9% in the DRF group. No significant group × time interaction was observed (*p* > 0.05), and the between-group effect size for change in body fat percentage was small (Cohen’s d = 0.35).

### 3.2. Fat Mass, Fat-Free Mass and Fat-Free Mass Index

Similarly, fat mass significantly declined over time in both groups (*p* < 0.001). The CCR group reduced fat mass by 9.30 ± 2.77 kg, while the DRF group reduced fat mass by 9.21 ± 2.63 kg. The difference in fat-mass loss between groups was negligible (*p* > 0.05), with a trivial effect size (Cohen’s d = 0.03), indicating virtually identical reductions in adipose tissue.

Fat-free mass demonstrated a significant main effect of time (*p* < 0.05). Participants in the CCR group increased fat-free mass by 1.22 ± 1.09 kg, whereas the DRF group increased fat-free mass by 2.40 ± 1.32 kg over the intervention period. Although the group × time interaction did not reach statistical significance (*p* = 0.08), the observed between-group difference corresponded to a moderate effect size favoring the DRF group (Cohen’s d = 0.98). This finding suggests a nonsignificant trend toward greater preservation of lean mass with the incorporation of structured diet breaks and should be interpreted cautiously.

Fat mass index (FMI) significantly decreased over time in both groups, reflecting meaningful reductions in adiposity (*p* < 0.001). The CCR group exhibited a decrease in FMI of −3.49 kg·m^−2^, while the DRF group demonstrated a comparable reduction of −3.41 kg·m^−2^. The between-group difference in FMI change was not statistically significant (*p* > 0.05) and corresponded to a trivial effect size, indicating virtually identical reductions in size-adjusted adiposity between dietary strategies. These findings are consistent with the observed changes in absolute fat mass and further support that both interventions produced equivalent improvements in adiposity when normalized to body size.

### 3.3. Energy Intake and Macronutrient Distribution

Mean daily caloric intake differed significantly between groups over the 12-week intervention (*p* < 0.01). Participants in the DRF group consumed 1562 ± 132 kcal·day^−1^, which was approximately 124 kcal·day^−1^ greater than the CCR group (1438 ± 86 kcal·day^−1^) due to the inclusion of three structured diet-break weeks. A significant group × week interaction was observed (*p* < 0.001), reflecting higher intake during diet-break weeks in the DRF group as shown in Table 1.

Protein intake was maintained at approximately 1.2 g·kg^−1^·day^−1^ in both groups, with no significant between-group differences (*p* > 0.05). Carbohydrate intake was higher in the DRF group (*p* < 0.05), whereas fat intake did not differ significantly between groups as shown in Table 2. These findings indicate that incorporating structured diet breaks allows for greater caloric and carbohydrate intake without compromising fat-loss outcomes, potentially supporting diet sustainability and lean mass preservation.

#### Dietary Adherence and Nutritional Compliance

Participants in the Diet Refeed group demonstrated high levels of adherence, with a mean dietary adherence rate of 91.36%, compared to 88.43% in the CCR group. Similarly, protein intake compliance was greater in the Diet Refeed group (93.07%) than in the CCR group (87.93%).

The percentage of days meeting prescribed caloric targets also favored the Diet Refeed group, which achieved 89.36%, whereas the CCR group achieved 85.57%.

### 3.4. Energy Balance and Adherence to Caloric Restriction

Changes in body composition indicated a sustained negative energy balance in both groups, with a significantly greater deficit observed in the continuous caloric restriction (CCR) group. Mean daily change in energy stores (ΔES) was −1005 ± 515 kcal·day^−1^ in the CCR group compared with −690 ± 120 kcal·day^−1^ in the diet refeed (DRF) group. This difference was statistically significant (*p* = 0.041), indicating a greater achieved energy deficit in the CCR condition. These findings provide an objective estimate of adherence independent of self-reported dietary intake, demonstrating that although both interventions produced meaningful energy deficits, the CCR group achieved a significantly greater magnitude of caloric restriction over the 12-week intervention.

## 4. Discussion

To our knowledge, this study is the first to examine a dietary strategy incorporating periodic carbohydrate refeeds and structured diet breaks during caloric restriction in women with obesity. The primary finding was that both continuous caloric restriction (CCR) and the diet-refeed with diet-break approach (DRF) produced significant reductions in fat mass and adiposity over 12 weeks, with no statistically significant differences between groups. These results are consistent with current clinical guidelines and a substantial body of literature demonstrating that overall energy deficit, rather than the temporal pattern of restriction, is the primary determinant of fat loss [1,2,3,4,10,11,12,13,15,16,17].

The present findings should therefore be interpreted in light of differences in achieved energy deficit between groups. Using a body composition–based approach consistent with the CALERIE trial [20], the CCR group demonstrated a significantly greater magnitude of caloric restriction compared to the refeed group, as evidenced by a larger daily energy deficit (−1005 ± 515 vs. −690 ± 120 kcal·day^−1^, *p* = 0.041). This objective method circumvents limitations of self-reported dietary intake, particularly underreporting, which is common in caloric restriction studies. Accordingly, differences in adherence, reflected in achieved energy deficit, may partially explain between-group differences in outcomes and should be considered when interpreting the physiological responses to each dietary strategy. This difference occurred despite similar reductions in fat mass between groups, reinforcing that total energy deficit rather than dietary pattern per se remains the primary driver of fat loss.

Both groups experienced comparable reductions in fat mass (approximately 9 kg), and no significant group × time interaction was observed for adiposity outcomes. These findings closely align with large randomized trials comparing intermittent and continuous energy restriction, including the HELENA trial and work by Sundfør et al., which reported similar fat loss and body-weight reductions when total caloric intake was broadly matched [15,16]. Together, these data indicate that incorporating planned periods of energy balance does not impair fat-loss efficacy but also does not clearly enhance it.

Notably, emerging evidence suggests that beyond total caloric intake, the temporal distribution of energy intake may influence metabolic outcomes. Studies examining meal frequency under conditions of matched caloric intake have demonstrated that altering the number and timing of meals can impact metabolic markers, including insulin dynamics and glycemic control, independent of changes in body weight [21,22,23]. While distinct from the intermittent restriction model employed in the present study, this body of literature highlights the broader concept that dietary patterning—including timing and frequency—may contribute to metabolic regulation and warrants consideration when interpreting dietary intervention effects.

Adherence data provide additional context for interpreting the present findings. The DRF group exhibited consistently higher adherence across all measured outcomes, including dietary adherence, protein intake compliance, and caloric target attainment. While both groups demonstrated generally high compliance, the CCR group showed modestly lower values across all metrics, suggesting slightly reduced consistency in meeting prescribed dietary targets. Greater variability was observed in the DRF group, particularly in caloric target attainment, likely reflecting the cyclical nature of refeeds and diet breaks. These findings suggest that intermittent strategies may enhance overall adherence for some individuals, potentially due to increased dietary flexibility and psychological relief from continuous restriction.

With respect to fat-free mass (FFM), the group × time interaction did not reach statistical significance (*p* = 0.08), despite a moderate effect size favoring DRF. Given the relatively small sample size (n = 30) and the inherent variability in FFM measurements, these findings should be interpreted as exploratory rather than confirmatory. The study was underpowered to detect small-to-moderate between-group differences in lean mass, and future studies with larger samples are needed.

The observed increases in FFM during a hypocaloric intervention, particularly in a non–resistance-trained cohort, require cautious interpretation. Body composition was assessed using air-displacement plethysmography, and FFM estimates derived from this method may be influenced by hydration status, glycogen storage, and associated water retention. This is especially relevant in the DRF group, where carbohydrate refeeds and maintenance-calorie periods may increase glycogen-associated water content. Accordingly, increases in FFM may reflect shifts in tissue hydration rather than true skeletal muscle hypertrophy.

Prior studies provide additional context. The MATADOR trial reported reduced metabolic adaptation with intermittent energy restriction compared with continuous restriction [13], while Campbell et al. demonstrated improved lean mass retention with carbohydrate refeeds in resistance-trained individuals [18]. However, key physiological variables such as resting energy expenditure, hormonal responses, and physical activity were not measured in the present study, limiting mechanistic interpretation and direct comparison.

Dietary protein intake was standardized at approximately 1.2 g·kg^−1^·day^−1^, exceeding the Recommended Dietary Allowance. Higher protein intakes are well established to support lean tissue retention during caloric restriction through stimulation of muscle protein synthesis and attenuation of proteolysis [24,25,26,27]. Consistent with current dietary guidelines, protein intakes in the range of 1.2–1.6 g·kg^−1^·day^−1^ are associated with improved body composition outcomes in individuals with overweight or obesity undergoing energy restriction [28], which may have contributed to the modest preservation of FFM observed in both groups.

From a clinical perspective, these findings suggest that structured diet breaks and carbohydrate refeeds can be implemented without compromising fat loss. Adherence patterns indicate that intermittent strategies may enhance sustainability, even in the absence of superior body composition outcomes. However, given the study limitations, including small sample size, reliance on self-reported dietary intake, lack of objective physical activity monitoring, and absence of metabolic or hormonal measurements, these findings do not support strong conclusions regarding the superiority of one dietary approach over another. CCR remains an effective and evidence-based strategy [2], while intermittent approaches may offer practical alternatives based on individual preference and adherence.

Several limitations should be acknowledged. First, the study was underpowered to detect small-to-moderate between-group differences in lean mass outcomes, which are inherently variable and typically require larger sample sizes for adequate statistical power. Second, dietary intake and adherence were assessed using self-reported methods, which are prone to systematic bias, including underreporting of energy intake. Consequently, reported caloric intake may not accurately reflect true energy consumption, and unmeasured differences in adherence between groups could have influenced the observed outcomes. Although measures of dietary adherence and compliance were included, objective validation using biomarkers (e.g., doubly labeled water, nitrogen balance, or respiratory quotient) was not performed. Finally, the wide age range of participants introduces additional heterogeneity in physiological responses to diet and training, which may limit the generalizability of the findings to more age-specific populations.

Relatedly, the absence of objective measures of energy balance limits interpretation of the prescribed versus achieved caloric restriction. In free-living conditions, actual energy deficit is more accurately reflected by changes in body composition rather than reported intake alone, and therefore true energy and protein balance cannot be definitively determined in the present study. Future investigations should incorporate objective measures of energy expenditure and nutrient balance to improve precision.

Additional limitations include the lack of assessment of menopausal status and hormonal contraceptive use, both of which may influence body composition and metabolic responses. Physical activity and resistance training were not standardized or objectively monitored, potentially introducing additional variability in lean mass outcomes. Furthermore, body composition was assessed via air-displacement plethysmography, which may be influenced by hydration status and glycogen-related water shifts. Finally, the relatively short intervention duration limits inference regarding longer-term metabolic adaptation and sustainability.

Taken together, these limitations constrain interpretation of the results and highlight the need for larger, well-controlled trials incorporating objective measures of energy intake, expenditure, and adherence.

## 5. Conclusions

In conclusion, both continuous caloric restriction and a dietary strategy incorporating carbohydrate refeeds and structured diet breaks produced meaningful reductions in fat mass in women with obesity. While a nonsignificant trend toward greater preservation of fat-free mass was observed with the intermittent approach, this finding requires confirmation in larger, adequately powered studies. Overall, these findings support the flexibility of dietary strategies in achieving fat loss and underscore the importance of adherence in determining outcomes.

## Figures and Tables

**Table 1:** Baseline participant characteristics by intervention group.

Variable	CCR (n = 15)	DRF (n = 15)
Age (years)	48.2 ± 10.1	51.7 ± 9.4
Height (cm)	163.3 ± 5.4	164.3 ± 5.8
Body mass (kg)	108.0 ± 13.1	109.0 ± 12.6
BMI (kg·m^−2^)	40.6 ± 4.5	40.4 ± 4.3
Body fat (%)	45.6 ± 4.8	44.5 ± 4.2

Note. Values are presented as mean ± standard deviation. No significant differences were observed between groups at baseline (*p* > 0.05 for all variables). CCR = continuous caloric restriction; DRF = diet refeed.

**Table 2:** Comprehensive Summary of Body Composition, Dietary Intake, and Adherence Outcomes.

Outcome	CCR (Mean ± SD)	DRF (Mean ± SD)	Statistical Interpretation
Body Weight Change (kg)	−7.53 ± 1.91	−6.80 ± 1.77	Significant ↓ over time; no between-group difference
Body Fat % Change (%)	−6.7 ± 2.1	−6.0 ± 1.9	Significant ↓ over time; small between-group effect
Fat Mass Change (kg)	−9.30 ± 2.77	−9.21 ± 2.63	Significant ↓; virtually identical between groups
Fat Mass Index Change (kg·m^−2^)	−3.49	−3.41	Significant ↓; consistent reductions in adiposity
Fat-Free Mass Change (kg)	+1.22 ± 1.09	+2.40 ± 1.32	Significant ↑ over time
Daily Energy Intake (kcal·day^−1^)	1435 ± 85	1555 ± 135 *	DRF higher intake due to diet breaks
Protein Intake (g-day)	118 ± 9	121 ± 8	No significant difference (matched intake)
Carbohydrate Intake (g-day)	165 ± 21	198 ± 29 *	Higher in DRF
Fat Intake (g-day)	48 ± 8	56 ± 10 *	Higher in DRF
Dietary Adherence Rate (%)	88.43	91.36	Higher adherence in DRF
Protein Intake Compliance (%)	87.93	93.07	Higher compliance in DRF

Note. Summary of body composition changes, dietary intake, and adherence outcomes in continuous caloric restriction (CCR) and diet refeed (DRF) groups over the 12-week intervention. Values are presented as mean ± standard deviation. Arrows indicate direction of change over time (↑ = increase; ↓ = decrease), while an asterisk denotes a statistically significant change (*p* < 0.05) within or between groups. CCR = continuous caloric restriction; DRF = diet refeed.

## Data Availability

The raw data supporting the conclusions of this article will be made available by the authors on request. The data are not publicly available due to ethical and privacy restrictions related to the protection of participant confidentiality.
